# Comparative Outcomes of Conventional Bardach Two-Flap Palatoplasty Versus Modified Alveolar Ridge/Crevicular Incision Technique in Isolated Cleft Palate Repair: A Prospective Observational Study

**DOI:** 10.7759/cureus.97428

**Published:** 2025-11-21

**Authors:** Saikrishna Degala, Amey Hariani

**Affiliations:** 1 Department of Oral and Maxillofacial Surgery, Jagadguru Sri Shivarathreeshwara (JSS) Dental College and Hospital, Mysuru, IND

**Keywords:** cleft palate, flap, incision, palatoplasty, prospective

## Abstract

Background

Isolated cleft palate (CP) disrupts the feeding, speech, and social development of young children. Standard Bardach two-flap palatoplasty effectively closes the defect but can leave large areas of exposed bone along the sides, particularly in wider clefts. The exposed bone may lead to scar formation and restricted jaw growth. A modified version of the two-flap technique uses incisions along the alveolar ridge or within the gum crevice to mobilize tissue, minimize exposed bone, and achieve closure without tension. This study compared the operative efficiency, postoperative complications, and functional outcomes between conventional and modified techniques in pediatric patients with isolated CP.

Methodology

This prospective, observational study was conducted over a period of 24 months. In total, 24 children aged 12-24 months with Veau I/II isolated CP were divided into the following two groups (n = 12 each): Group A underwent conventional Bardach two-flap palatoplasty, and Group B underwent the modified technique with alveolar ridge/crevicular incisions. All surgeries were performed under general anesthesia with intravelar veloplasty and layered closure using 4-0 Vicryl sutures. The outcomes assessed included operative time, tension-free closure, fistula formation (Pittsburgh classification), nasal regurgitation, uvular morphology, and deciduous teeth eruption patterns. Follow-up was extended to 18 months. Data were analyzed using Fisher’s exact test and the independent t-test (p < 0.05).

Results

Demographic characteristics were comparable between the groups. The mean operative time was 135 minutes (conventional) versus 141.67 minutes (modified) (p = 0.387). Tension-free closure was achieved in eight (66.67%) conventional and 12 (100%) modified cases (p = 0.121). No fistulas or nasal regurgitations occurred in either group. Two (16.67%) patients per group had a minor delay in deciduous tooth eruption (p = 1.0). Uvular morphology was satisfactory in six (50%) conventional and eight (66.67%) modified cases (p = 0.558). All differences were not statistically significant.

Conclusions

Both conventional and modified two-flap palatoplasty techniques yielded equivalent surgical and functional outcomes, with minimal morbidity in Veau I/II isolated CP repair. The modified approach tended toward better mobilization and tension-free closure in wider defects without increasing the operative time or complications. Larger randomized trials with long-term cephalometric and speech evaluations are recommended to confirm these clinical benefits.

## Introduction

Orofacial clefts represent the most prevalent congenital craniofacial anomalies worldwide, with isolated cleft palate (CP) occurring in approximately 1 in 700 live births globally; orofacial clefts have been estimated at 4.6 million [1,2]. In India, the estimated prevalence rate/100,000 for orofacial cleft in 2016 was 33.27 for males, 31.01 for females, and 32.18 combined for both sexes [3]. These anomalies profoundly affect craniofacial development, speech, feeding, and psychosocial well-being, necessitating early surgical intervention to restore anatomical integrity and functional competence [4]. Palatoplasty, the surgical reconstruction of the cleft palate, aims to achieve tension-free closure of the nasal and oral layers, reconstruct the velopharyngeal musculature, and minimize postoperative complications such as fistula formation, maxillary growth disturbance, and velopharyngeal insufficiency [5].

Bardach two-flap palatoplasty, a cornerstone technique for CP repair, involves lateral releasing incisions at the junction of the hard palate and alveolar ridge, elevation of bipedicled mucoperiosteal flaps, and layered midline closure [6]. Although effective, this method may result in extensive lateral denuded bone, particularly in wider clefts, potentially increasing the risk of scar contracture and maxillary hypoplasia. A modified two-flap palatoplasty utilizing an alveolar ridge or crevicular incision has been proposed to enhance flap mobilization, reduce bone exposure, and facilitate tension-free closure in challenging cases [7]. Despite promising clinical observations, comparative evidence of operative efficiency, postoperative morbidity, and functional outcomes remains limited, with no randomized trials directly contrasting these approaches in isolated CP.

This study aimed to compare the surgical outcomes and complications of Bardach’s conventional two-flap palatoplasty with a modified two-flap technique utilizing alveolar ridge or crevicular incisions in patients with Veau I and II isolated CP. The specific objectives were to evaluate the operative duration and ease of flap mobilization, assess postoperative complications including fistula formation and nasal regurgitation, compare uvular morphology and deciduous tooth eruption patterns, and determine technical advantages in achieving tension-free closure of wider clefts. The null hypothesis posited that there would be no statistically significant difference in surgical outcomes, complication rates, or functional results between conventional Bardach’s two-flap palatoplasty and the modified alveolar ridge/crevicular incision technique.

## Materials and methods

Study design and setting

This study was designed as a prospective, comparative, observational study to evaluate and compare the outcomes of two surgical techniques for repairing isolated cleft palate in pediatric patients. The CP procedures were performed in the department according to each surgeon’s clinical preference and technique of choice. The investigators systematically observed and recorded relevant intraoperative and postoperative parameters without influencing the surgical decision-making process. The study was conducted at the Department of Oral and Maxillofacial Surgery, Jagadguru Sri Shivarathreeshwara (JSS) Dental College and Hospital, Mysuru, Karnataka, India. Data collection spanned 24 months from the initiation of patient recruitment to the completion of the minimum follow-up period. Ethical approval was obtained from the JSS Dental College and Hospital Institutional Ethics Committee (approval number: 18/2023). Written informed consent was obtained from the parents or legal guardians of all participating patients before enrolment, ensuring that they were fully informed about the study procedures, potential risks, benefits, and their right to withdraw at any time without affecting standard care.

Sample size calculation

A sample size of 20 was calculated using G*Power software (version 3.1.9.2, Heinrich Heine University, Düsseldorf, Germany). The calculation was based on a large effect size of 0.91, as determined from a previous study by Peyvasteh et al. [8] comparing cleft width (mean 13 mm and 15 mm) between the two palatoplasty techniques. The study was powered at 80% with a 5% significance level. The sample size formula was as follows:

\begin{equation} n = \frac{(Z_{1-\alpha/2} + Z_{1-\beta})^2 \cdot \sigma^2}{(\mu_1 - \mu_2)^2} \label{eq:sample_size} \end{equation}

where n = sample size, Z₁₋α/₂ = confidence interval at 95% (1.96), Z₁₋β = power of study at 80% (0.84), σ = pooled standard deviation, and μ₁ - μ₂ = mean difference.

A minimum of 20 patients (10 in each group) were required. Considering a 10% non-response rate, a total of 24 patients (12 per group) were included to ensure a more robust analysis of the study.

Eligibility

Patients were eligible for inclusion if they presented with isolated CP classified as Veau I or Veau II [9], were aged between 12 and 24 months at the time of surgery to align with the optimal developmental windows for palatoplasty, and were deemed medically fit for general anesthesia following multidisciplinary evaluations by pediatricians, otolaryngologists, and cardiologists. Exclusion criteria included syndromic CP, concurrent cleft lip, additional congenital anomalies affecting craniofacial development, or any contraindications to surgery such as active infections, severe malnutrition, or cardiopulmonary instability.

Methodology

**Figure 1:**
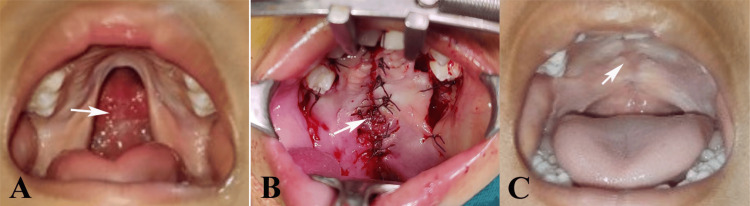
Conventional Bardach’s two-flap palatoplasty: (A) cleft palate, (B) suturing the approximated flaps, and (C) healed palate after 18 months. Original images of the patient from the study, used with parents’ consent.

The study involved 24 pediatric patients divided into the following two groups of six each: Group A consisted of patients who underwent conventional Bardach’s two-flap palatoplasty, and Group B consisted of patients who underwent modified two-flap palatoplasty using alveolar ridge or crevicular incisions. All procedures were performed under general anesthesia following oral intubation, and surgical exposure was achieved using a Dingman mouth retractor (Integra LifeSciences, Princeton, NJ, USA) (Figure 1).

**Figure 2:**
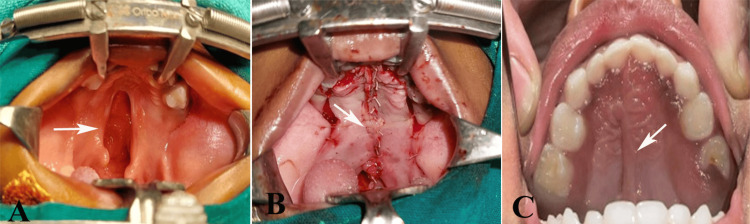
Modified two-flap palatoplasty using alveolar ridge or crevicular incisions: (A) cleft palate, (B) suturing of approximated flaps, and (C) healed palate after 18 months. Original images of the patient from the study, used with parents’ consent.

For the modified technique in Group B, incisions were made along the cleft margins to circumferentially separate the nasal and oral mucosa, followed by lateral releasing incisions placed strategically along the crest of the alveolar ridge in edentulous areas or within the dental sulcus when dentition was present, rather than at the junction between the alveolar ridge base and hard palate. Mucoperiosteal flaps were then elevated bluntly in the subperiosteal plane from the maxillary and palatine bones, with care taken to preserve the greater palatine neurovascular bundles bilaterally. Soft palate repair incorporated a standard linear closure with intravelar veloplasty to re-approximate the muscle layer, and vomer flaps were elevated when necessary to facilitate closure (Figure 2). The nasal mucosa was sutured first using 4-0 Vicryl absorbable sutures (Ethicon, New Brunswick, NJ, USA), followed by midline approximation of the mobilized mucoperiosteal flaps using the same suture material. In the posterior region, layered closure of the nasal mucosa, palatal musculature, and oral mucosa was completed using 4-0 Vicryl sutures to ensure tension-free and watertight closure [7].

For Group A, conventional Bardach’s two-flap palatoplasty followed the standard protocol [6], involving lateral incisions at the palate-alveolus junction, flap elevation, muscle realignment via intravelar veloplasty, and layered suturing with 4-0 Vicryl sutures, without alveolar ridge modification. Intraoperative parameters such as operative time were recorded from incision to closure. Postoperative care included monitoring in a recovery unit; administration of analgesics and antibiotics as per institutional protocols; and scheduled follow-up visits at one week, one month, three months, six months, and up to 18 months to assess healing and complications.

Outcome assessments

Postoperative outcomes were evaluated clinically using a combination of objective measurements and standardized classifications to comprehensively compare the two techniques. Operative time was measured in minutes from the start of incision to final closure, and means and standard deviations were calculated for each group to assess procedural efficiency. Fistula formation was monitored at all follow-up visits using the Pittsburgh fistula classification system, which categorizes fistulas anatomically into seven types based on location (such as Type I at the uvula and Type IV in the hard palate), allowing for precise identification of any oronasal communications that could lead to functional issues such as nasal regurgitation or speech distortions [10]. This classification system is freely available for both clinical and research purposes.

Disturbance in the eruption of deciduous teeth was assessed in patients for 18 months using a grading system for tooth emergence, categorizing crown exposure into a four-tier scale based on thirds (such as Grade C for partial emergence and Grade E for full eruption), and comparisons were made against ideal eruption timelines and between groups to detect any technique-related delays or disruptions [11]. The grading system is freely available for clinical and research use as the article is open access under a Creative Commons license (CC BY-NC-ND 4.0).

Uvular morphology was evaluated at least six months postoperatively using a classification system that grades the uvula as absent, small remnant, bifid, deviated, or satisfactory [12]. The uvular morphology classification system is freely available for clinical and research use, with only a proper citation required. Additional assessments included clinical observation of nasal regurgitation, overall palatal healing, and surgeon-reported ease of flap mobilization.

Statistical analysis

Data were analyzed using SPSS software version 23 (IBM Corp., Armonk, NY, USA). Categorical variables were summarized as frequencies and percentages, while continuous variables, such as age and operative time, were expressed as means and standard deviations. The Shapiro-Wilk test confirmed that the data followed a normal distribution. Categorical variables were compared using Fisher’s exact test, whereas independent t-tests were used for continuous variables. Statistical significance was set at p-values <0.05.

## Results

The demographic analysis in Table 1 indicates no statistically significant differences between the conventional and modified groups in terms of sex distribution and age. The male and female proportions were comparable (p = 0.221), and the mean age was similar (p = 0.635). These findings suggest that the study groups were demographically balanced, minimizing bias related to sex or age in the subsequent analyses.

**Table 1:** Demographic characteristics of the study population. Numbers of males and females are presented as frequency (n) and percentage (%), whereas age is expressed as mean ± standard deviation (SD). P-values >0.05 denote no statistical significance, using Fisher’s exact test for sex and an independent t-test for age.

Demographic	Variables	Bardach’s two-flap palatoplasty	Modified two-flap palatoplasty	Test value	P-value
Sex	Male	10 (83.33%)	6 (50%)	1.5	0.221
	Female	2 (16.67%)	6 (50%)		
Age (months)	Mean ± SD	17.17 (4.62)	19.33 (9.77)	0.48	0.635

The results showed that a higher proportion of patients in the modified group received tension-free sutures (n = 12, 100%) than those in the conventional group (n = 8, 66.67%), although this difference was not statistically significant (p = 0.121). The mean operative time was also slightly longer in the modified group (141.67 vs. 135 minutes); however, the difference was not statistically significant (p = 0.387). These results suggested no significant operative advantage between the techniques for these parameters (Table 2).

**Table 2:** Comparison of operative parameters between the two surgical techniques. Data for tension-free suture are presented as frequency (n) and percentage (%), whereas operative time is expressed as mean ± standard deviation (SD). P-values >0.05 denote no statistical significance, using Fisher’s exact test for tension-free suture and an independent t-test for operative time.

Operative parameters	Variables	Bardach’s two-flap palatoplasty	Modified two-flap palatoplasty	Test value	P-value
Tension-free suture	Yes	8 (66.67%)	12 (100%)	2.4	0.121
	No	4 (33.33%)	0 (0%)		
Operative time (minutes)	-	135 (11.85)	141.67 (13.66)	0.9	0.387

The results showed that no cases of fistula or nasal regurgitation occurred in either the conventional or modified groups. Abnormal deciduous teeth eruption was observed in two (16.67%) patients in both groups. Statistical analysis revealed no significant differences in any complications (all p = 1). This indicates similar safety profiles between the techniques, with a low incidence of adverse events, and no operative group demonstrating a significant risk for the complications studied (Table 3).

**Table 3:** Comparative analysis of complications between groups. Data are presented as frequency (n) and percentage (%). P-values >0.05 denote no statistical significance, using Fisher’s exact test.

Complications	Variable	Bardach’s two-flap palatoplasty	Modified two-flap palatoplasty	Test value	P-value
Oronasal fistula	Yes	0 (0%)	0 (0%)	0.0	0.99
	No	12 (100%)	12 (100%)		
Nasal regurgitation	Yes	0 (0%)	0 (0%)	0.0	0.99
	No	12 (100%)	12 (100%)		
Abnormal deciduous teeth eruption	Yes	2 (16.67%)	2 (16.67%)	0.0	0.99
	No	10 (83.33%)	10 (83.33%)		

The results showed comparable outcomes in terms of uvula appearance in the conventional and modified groups. In the conventional group, six (50%) patients displayed a bud-like uvula, while six (50%) patients achieved a satisfactory appearance. The modified group had four (33.33%) patients with a bud-like uvula, and eight (66.67%) were satisfactory cases. The differences observed between the groups were not statistically significant (p = 0.558). Thus, both techniques yielded similar esthetic results in uvula restoration, with no clear advantage of either method in improving postoperative appearance (Table 4). Therefore, our results failed to reject the null hypothesis.

**Table 4:** Comparison of outcome between study groups. Data are presented as frequency (n) and percentage (%). P-values >0.05 denote no statistical significance, using Fisher’s exact test.

Results	Variables	Bardach’s two-flap palatoplasty	Modified two-flap palatoplasty	Test value	P-value
Uvula appearance	Bud	6 (50%)	4 (33.33%)	0.34	0.558
	Satisfactory	6 (50%)	8 (66.67%)		

## Discussion

The present prospective comparative study evaluated the surgical outcomes between conventional Bardach two-flap palatoplasty and a modified variant employing alveolar ridge or crevicular incisions in pediatric patients with Veau I and II isolated CP. Intraoperative findings revealed comparable efficiency between the techniques, with operative durations closely matched. The marginal increase in time for the modified method likely stems from precise incision placement along the alveolar crest or sulcus, which requires meticulous dissection to preserve the periosteal integrity and neurovascular structures. This is supported by the findings of Sommerlad [13], who noted that strategic lateral releases in two-flap variants enhance mobilization without proportional time escalation as subperiosteal elevation remains streamlined.

Similarly, the higher incidence of tension-free closures in the modified group, albeit non-significant, supports the biomechanical advantage of relocating the releasing incisions away from the palate-alveolus junction. By minimizing raw bone exposure at the hard palate base, this adjustment reduces contractile forces on maxillary segments, similar to findings in von Langenbeck repairs adapted for wider clefts [14]. Peyvasteh et al. [8] previously demonstrated that alveolar extensions facilitate greater flap advancement in clefts exceeding 12 mm, corroborating the ease of mobilization reported by the operating surgeon.

Postoperative morbidity profiles were remarkably low across both arms, underscoring the safety of two-flap palatoplasty irrespective of incision modification. Zero fistula rates challenge historical benchmarks, where Peyvasteh et al. [8] noted the presence of 33.3% oronasal fistulas using the Bardach technique in isolated CP. Although similar findings were reported in a previous study, 40-50% spontaneous healing of wound dehiscence was noted, which was attributed to low tension on the closure line achieved using a delicate surgical technique and relaxing incisions [15]. The absence of oronasal fistulas in our study may be attributed to rigorous layered closure with intravelar veloplasty, which reconstructs the levator sling and reinforces velopharyngeal competence, as validated in long-term cohorts by Yang et al. [16].

The solitary instances of deciduous tooth eruption disturbance per group aligned with minimal periosteal disruption in both methods: conventional incisions, though more lateral, spared apical regions, while modified crevicular placements avoided gingival margins in dentate cases. Elkalla et al. [17] reported significantly faster tooth eruption with alveolar extension palatoplasty than with the Bardach technique. Functional equivalence was extended to velopharyngeal outcomes, with the absence of nasal regurgitation reflecting effective muscle reorientation. Uvular morphology, a surrogate for soft palate esthetics and function, showed no preferential restoration, consistent with Rossell-Perry et al. [18], who conducted a prospective, randomized, double-blind clinical trial and found that a specific method for uvular repair during primary palatoplasty did not significantly influence the final uvular bulk or appearance, despite variations in the midline closure technique.

Collectively, these results fail to reject the null hypothesis. The modified technique’s potential for reduced denuded bone, implied by enhanced mobilization, mirrors innovations in Furlow double-opposing Z-plasty hybrids [19], yet without randomized evidence, its superiority remains unsubstantiated. Murthy et al. [20] noted the formation of an oronasal fistula as a postoperative complication after a two-flap palatoplasty procedure and reported the presence of a fistula in eight (2.4%) cases. Khetpal et al. [7] documented the occurrence of oronasal fistula in two (7%) patients using a modified surgical approach. They additionally noted that the identified fistulae were situated along the midline, indicating a possible tissue deficiency, which could theoretically be ameliorated through the implementation of larger flaps. No significant complications were reported at the dental margin or concerning the patient’s dentition, implying that the incision made at this site did not yield any detrimental effects.

From a clinical standpoint, both methods offer reliable, low morbidity options for Veau I/II repairs, with the modified approach particularly advantageous in wider clefts (>15 mm) where conventional release risks excessive scarring. Surgeons may selectively adopt crevicular incisions to mitigate maxillary hypoplasia risks without compromising efficiency, especially in resource-constrained settings such as tertiary Indian centers. This flexibility enhances personalized care and potentially improves velopharyngeal outcomes in challenging anatomies.

Limitations include the small sample size and observational design prone to selection bias despite the clinical rationale. Follow-up, capped at 18 months, precludes the assessment of midfacial growth or speech articulation maturation, necessitating long-term studies. Additionally, single-surgeon execution, while ensuring consistency, limits generalizability across varied expertise. Future randomized trials with larger cohorts and cephalometric/speech endpoints are warranted to delineate these subtle advantages.

## Conclusions

In this prospective study, conventional Bardach two-flap palatoplasty and modified alveolar ridge/crevicular incision technique demonstrated equivalent surgical outcomes in Veau I/II isolated CP repair. Both methods achieved tension-free closure, low complication rates, and a comparable uvular morphology without fistula or nasal regurgitation. The operative times and deciduous teeth eruption patterns were similar, failing to reject the null hypothesis. The modified technique offers potential advantages in wider clefts by reducing the denuded bone, although it is not statistically superior. Both approaches are safe and effective and support individualized surgical selection. Long-term randomized trials are needed to assess the growth and speech outcomes.
